# Using Intrinsic Flavoprotein and NAD(P)H Imaging to Map Functional Circuitry in the Main Olfactory Bulb

**DOI:** 10.1371/journal.pone.0165342

**Published:** 2016-11-30

**Authors:** Cedric R. Uytingco, Adam C. Puche, Steven D. Munger

**Affiliations:** 1 Department of Anatomy and Neurobiology, University of Maryland School of Medicine, Baltimore, Maryland, United States of America; 2 Program in Neuroscience, University of Maryland School of Medicine, Baltimore, Maryland, United States of America; 3 Center for Smell and Taste, University of Florida, Gainesville, Florida, United States of America; 4 Department of Pharmacology and Therapeutics, University of Florida, Gainesville, Florida, United States of America; 5 Department of Medicine, Division of Endocrinology, Diabetes and Metabolism, University of Florida, Gainesville, Florida, United States of America; University of Queensland, AUSTRALIA

## Abstract

Neurons exhibit strong coupling of electrochemical and metabolic activity. Increases in intrinsic fluorescence from either oxidized flavoproteins or reduced nicotinamide adenine dinucleotide (phosphate) [NAD(P)H] in the mitochondria have been used as an indicator of neuronal activity for the functional mapping of neural circuits. However, this technique has not been used to investigate the flow of olfactory information within the circuitry of the main olfactory bulb (MOB). We found that intrinsic flavoprotein fluorescence signals induced by electrical stimulation of single glomeruli displayed biphasic responses within both the glomerular (GL) and external plexiform layers (EPL) of the MOB. Pharmacological blockers of mitochondrial activity, voltage-gated Na^+^ channels, or ionotropic glutamate receptors abolished stimulus-dependent flavoprotein responses. Blockade of GABA_A_ receptors enhanced the amplitude and spatiotemporal spread of the flavoprotein signals, indicating an important role for inhibitory neurotransmission in shaping the spread of neural activity in the MOB. Stimulus-dependent spread of fluorescence across the GL and EPL displayed a spatial distribution consistent with that of individual glomerular microcircuits mapped by neuroanatomic tract tracing. These findings demonstrated the feasibility of intrinsic fluorescence imaging in the olfactory systems and provided a new tool to examine the functional circuitry of the MOB.

## Introduction

**Fig 1:**
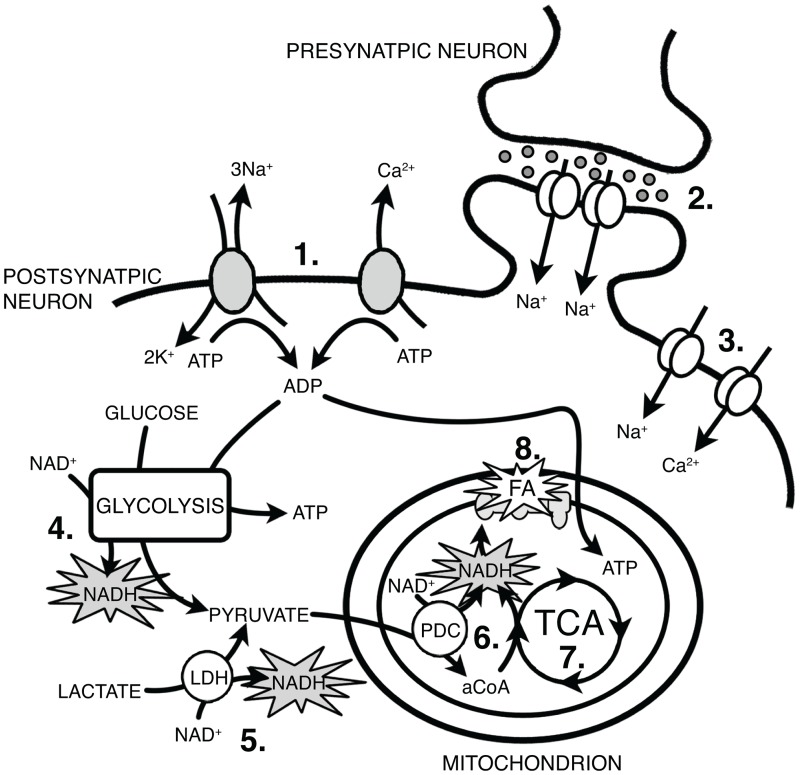
Principles of intrinisic flavoprotein and NAD(P)H fluorescent imaging. Instrinsic flavoprotein (FA) and NAD(P)H fluorescent signals are attributed to mitochondrial activity. Changes in fluorescence are correlated with neuronal activity through increases in metabolic demand resulting from ionic pumps and synaptic activity. (1) Na^+^/K^+^ and Ca^2+^ ATPases, (2) synaptic NMDA and AMPA receptors, (3) voltage-gated Na^+^ and Ca^2+^ channels, (4) glycolysis, (5) lactate dehydrogenase (LDH), (6) pyruvate dehydrogenase complex (PDC), acetyl-coenzyme A (aCoA), (7) tricarboxylic acid cycle (TCA), (8) electron transport chain.

Functional optical imaging has been a valuable tool for examining the spatiotemporal connectivity of neuronal circuits. There are a variety of optical techniques available to assess neuronal activity, including externally applied and/or genetically encoded indicators for Ca^2+^, voltage and pH, as well as changes in endogenous hemodynamic and intrinsic fluorescence signals. Recently, there has been renewed interest in utilizing intrinsic fluorescence signals given the improved technology and accessibility of the technique, which does not require external dyes or genetically modified cells. Intrinsic fluorescence imaging relies on the fluorescent properties of either oxidized flavoproteins (excitation: 430–480 nm/emission: 520–590 nm) or reduced nicotinamide adenine dinucleotide (phosphate) [NAD(P)H] (excitation: 340–360 nm/emission: 430–450 nm) [1–3]. Changes in flavoprotein and NAD(P)H fluorescence signals are associated with mitochondrial activity, where both molecules participate in the electron transport chain leading to ATP synthesis (Fig 1). Neuronal activity and metabolism are strongly coupled in neurons due to the high energetic demands of Na^+^/K^+^-ATPase and Ca^2+^-ATPase following action potential generation and synaptic signaling [4, 5] (Fig 1). Thus, increases in action potential firing rates strongly enhance intrinsic flavoprotein and NAD(P)H fluorescence signals. Both imaging techniques have proven to be highly stable, sensitive and specific markers for neuronal activation both *in vivo* and *in vitro* [5–8]. Intrinsic flavoprotein imaging in particular has been used to map the functional connectivity of the auditory [8, 9], thalamocortical [7, 10], and cerebellar systems [1, 5, 6]. Here we tested whether this functional imaging approach could be applied to imaging the structure function of the main olfactory bulb (MOB) circuitry.

The MOB receives afferent input from olfactory sensory neurons (OSNs) within the main olfactory epithelium. Upon entering the MOB, these OSN axons are reorganized such that axons from OSNs expressing the same odorant receptor coalesce to innervate one or a few glomeruli [11, 12]. Within these glomeruli, OSN axons synapse with juxtaglomerular interneurons and mitral/tufted cells (the projection neurons of the MOB) to form the glomerular circuit [11–17]. A number of tracing and functional imaging studies have provided important insights into the connectivity and complexity of the local glomerular circuit [18]; however, technical limitations have hindered our understanding of the functional connectivity of the circuit over long distances within the MOB. This study establishes intrinsic flavoprotein and NAD(P)H imaging as a novel tool for studying MOB functional circuit organization.

## Materials and Methods

### Animals and olfactory bulb slice preparation

All studies were approved by the University of Maryland, Baltimore IACUC committee. Animals (3–6 week old male and female C57BL6/J mice) were anesthetized with saturated isoflurane vapor and the olfactory bulbs surgically removed and immediately placed in 4°C oxygenated sucrose-artificial cerebrospinal fluid (sucrose-ACSF) containing 26 mM NaHCO_3_, 1 mM NaH_2_PO_4_, 3 mM KCl, 5 mM MgSO_4_, 0.5 mM CaCl_2_, 10 mM glucose, and 248 mM sucrose, equilibrated with 95% O_2_-5% CO_2_, pH 7.38. Horizontal slices (380–400 μm thick) were cut with a Leica VT1000 vibratome. Slices were incubated in oxygenated ACSF (containing 124 mM NaCl, 26 mM NaHCO_3_, 3 mM KCl, 1.25 mM NaH_2_PO_4_, 2 mM MgSO_4_, 2 mM CaCl_2_, and 15 mM glucose equilibrated with 95% O_2_-5% CO_2_, pH 7.4) at 30°C for 20–30 min then at room temperature (22°C) in ACSF for ~1 hr prior to use. For imaging sessions, individual slices were transferred to a custom recording chamber and perfused with ACSF maintained at a constant 30°C (Bipolar Temperature Controller, Norfolk, VA) at a rate of 2.5 ml/min.

In some experiments, drugs or other compounds were added to the ACSF solution perfused into the chamber. The pharmacological agent, substrate change or washouts were perfused into the chamber for 15 min prior to the start of each recording. Pharmacological agents used for the experiments include mitochondrial toxin diphenyleneiodium (DPI), voltage-gated Na^+^ channel toxin tetrodotoxin (TTX), AMPA receptor antagonist 2,3-dihydroxy-6-nitro-7-sulfamoyl-benzo[f]quinoxaline-2,3-dione (NBQX), NMDA receptor antagonist (2*R*)-amino-5-phosphonovaleric acid; (2*R*)-amino-5-phosphonopentanoate (AP-V), and GABA_A_ receptor antagonist 4-[6-imino-3-(4-methoxyphenyl)pyridazin-1-yl] butanoic acid hydrobromide (gabazine, GBZ). In some experiments, sodium lactate (15 mM) was added to the ACSF solution as a substitute for glucose.

### Stimulation protocol

Single target glomeruli on the medial aspect of the MOB were electrically stimulated at the apical border, within 10–20 μm of the olfactory nerve layer, using a pulled theta glass electrode with a 5 μm tip. Unless otherwise indicated, glomeruli received a train stimulus of 100 μA, 50 Hz (pulses/s), 1 ms pulse width, with a duration of 2 sec. This baseline stimulation parameter yielded the most robust and reproducible intrinsic response. For experiments examining the impact of stimulation parameters, the stimulation strength (10 μA to 100 μA), stimulation frequency (10 Hz to 50 Hz), or train duration (0.5 s to 6.0 s) were varied. All stimulation protocols were delivered through a Cygnus stimulus-isolating unit and driven by a Cygnus PG4000 digital stimulator.

### Intrinsic flavoprotein and NAD(P)H imaging

Images were collected using a Retiga EXi CCD camera (QImaging) attached to an epifluorescent microscope (Olympus BX51WI) equipped with a 10x water immersion objective lens (0.30 NA) or a 4x lens (0.13 NA) with a custom water immersion adaptor. Standard emission/excitation cubes optimal for flavoprotein (excitation: 430–500 nm; emission: 520–590 nm) or NAD(P)H (excitation: 300–400 nm; emission: 430–500 nm) were used. In order to limit potential phototoxic effects of 100W mercury lamp light exposure, neutral density filters were installed in the illumination path. For flavoprotein imaging, no filter was used for imaging at 4x, while a 0.3 optical density filter was used for imaging at 10x. To limit the more harmful 300–400 nm excitation light in NAD(P)H imaging, 0.3 and 0.6 optical density filters were used for imaging at 4x and 10x, respectively. All images were acquired through the Matlab Image Acquisition Toolbox (Mathworks, Matlab v2011b) at 2 frames/s (500 ms exposure) and 360x260 pixel (px) resolution (using 4x4 hardware binning). The images were subsequently processed and analyzed on a pixel-by-pixel basis using the Matlab Image Processing Toolbox (Mathworks, Matlab v2011b) and a script modified from Theyel and colleagues [19]. The first 10 pre-stimulus frames were used to determine the relative fluorescence intensity (ΔF/F) or [(F_n_–F_m_)/F_m_] for each recording trial. For each stimulus parameter or treatment condition, 3–5 repeated trials were averaged prior to signal quantification. Nonspecific background fluorescence and fluorescent bleaching were removed by subtracting non-stimulus ΔF/F recordings from the stimulus ΔF/F recordings. For all displayed images, the processed ΔF/F images were overlaid on top of the static raw fluorescent image. The pixel value for the ΔF/F images were scaled and cutoff at minimum 0% and maximum 4–5% ΔF/F for intrinsic flavoprotein imaging. For intrinsic NAD(P)H imaging, ΔF/F images were scaled and cutoff at minimum -4% and maximum 0% ΔF/F, represented in inverse colors.

### Data analysis

Flavoprotein and NAD(P)H signal analyses were performed after repeated recording averaging and background subtraction. Point region of interests (ROIs) were 8x8 px (20x20 μm) for recordings at 10x magnification. Glomerular layer (GL) ROIs were positioned at the center of the stimulated glomerulus. External plexiform layer (EPL) ROIs were directly below the glomerular ROI and midway between the apical and basal EPL borders. Signal amplitude, full duration at half-maximal (FDHM), time-to-peak, and time-to-trough were determined based on the GL and EPL ROI traces, using a custom MATLAB script. When available, the analyses were performed on both the stimulus-dependent light and dark phases. Unless otherwise specified, repeated measures analysis of variance (RM-ANOVA) was performed for all experimental conditions followed by Tukey pairwise comparison post-hoc test. Linear regression analysis was performed for experiments examining signal response relative to changes in stimulation strength, frequency or duration. All statistical analyses were performed in Sigmaplot (Sigmaplot v12) and GraphPad Prism (Prism v6.05) software. Vertical heat maps were generated using vertical ROIs at 8 px (20 μm) and 5 px (50 μm) widths for 10x and 4x magnifications, respectively. Horizontal heat maps were generated using horizontal ROIs midway to the depth of the GL and EPL at 8 px (20 μm) and 5 px (50 μm) widths for 10x and 4x magnifications, respectively. Signal full width at half-maximal (FWHM) was generated by averaging the horizontal ROI traces during the 2s stimulation period.

## Results

### Glomerular stimulation elicits translaminar spread of flavoprotein signals

**Fig 2:**
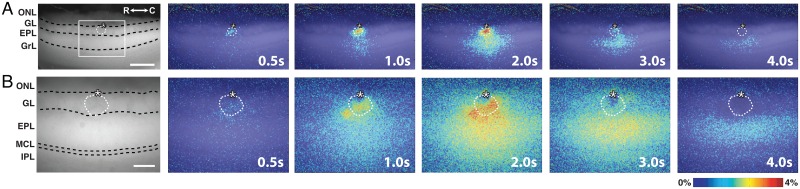
Intrinsic flavoprotein signal response in olfactory bulb upon electrical stimulation of canonical glomerulus. **(A)** (left) Resting light fluorescent image of a horizontal MOB slice under 4x magnification. (right) Representative overlaid, pseudo-colored ΔF/F frames showing the signal spread following initiation of train stimulation at 0s. Scale bar = 400 μm. **(B)** (left) Resting light fluorescent image of the demarcated region of the same slice in (A) at 10x magnification. (right) Representative overlaid, pseudo-colored ΔF/F frames following initiation of train stimulation at 0s. ONL, olfactory nerve layer; GL, glomerular layer; EPL, external plexiform layer; MCL, mitral cell layer; IPL, internal plexiform layer; GrL, granule cell layer; R, rostral; C, caudal. Asterisk, tip of the stimulating electrode. Targeted glomerulus indicated by dashed circle. Scale bar = 150 μm.

**Fig 3:**
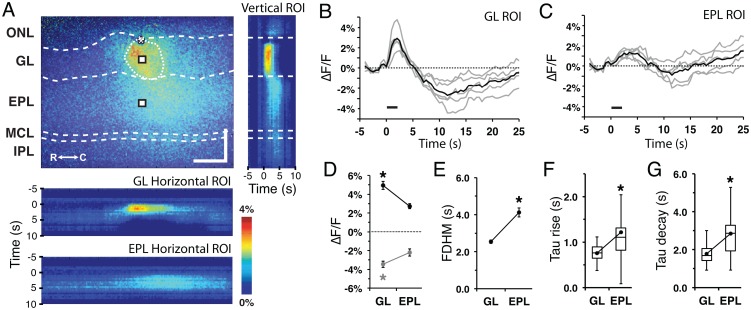
Flavoprotein signal profile in the glomerular and external plexiform layers. **(A)** (top) Flavoprotein response at 2.5s post-stimulus initiation. ONL, olfactory nerve layer; GL, glomerular layer; EPL, external plexiform layer; MCL, mitral cell layer; IPL, internal plexiform layer; GrL, granule cell layer; R, rostral; C, caudal. Asterisk, tip of the stimulating electrode. Targeted glomerulus indicated by dashed circle. Scale bars = 150 μm; (right) Vertical ROI heat map across the MOB layers exhibiting translaminar stimulus-dependent spread over time; (bottom) Horizontal ROI heat maps from the GL and EPL exhibiting stimulus-dependent lateral spread over time. **(B and C)** Representative ROI individual (gray) and mean (black) traces from the activated (B) glomerular and (C) EPL, indicated in (A). Line: stimulus train delivery and duration. **(D)** Mean signal amplitude of the light and dark phases between the GL and EPL areas. **(E)** Mean FDHM between the GL and EPL. **(F and G)** Flavoprotein signal (F) time-to-peak and (G) time-to-trough between the GL and EPL.

Stimulus-dependent flavoprotein responses from horizontal MOB slices were imaged using a standard CCD-equipped epifluorescence microscope that allowed simultaneous visualization of six distinct layers of the MOB: the olfactory nerve layer (ONL), glomerular layer (GL), external plexiform layer (EPL), mitral cell layer (MCL), internal plexiform layer (IPL), and granule cell layer (GrL) (Fig 2). Train stimulation (100 μA, 50 Hz, 2 s) of individual glomeruli evoked a relative increase in fluorescence (ΔF/F) signal visible at low (4x, field of view 2 mm: Fig 2A) and high (10x, field of view 800 μm; Fig 2B) magnifications. Due to the organization of the glomerular circuit and the distinct compartmentalization of the neurons involved in the circuit, analyses focused on signals in the GL and EPL (Fig 3). Vertical ROIs taken perpendicular to the MOB surface and centered on the stimulated glomerulus exhibited a stimulus-dependent signal that spread across the layers of the MOB over time (Fig 3A, right). Horizontal ROIs taken from the middle of the GL and EPL also exhibited lateral stimulus-dependent signal spread over time (Fig 3A, bottom). Point ROIs taken from the stimulated glomerulus and the EPL exhibited a rapid rise in ΔF/F signal (light phase), followed by a slow decrease in signal (dark phase) before returning back to baseline levels (Fig 3B and 3C). This biphasic response was similar to flavoprotein fluorescence responses observed in other brain regions [1, 7]. Quantification of the responses revealed differences in the signal characteristics between the GL and EPL. The amplitudes of both the GL light and dark phase responses were significantly greater than those in the EPL (light: F_(1,50)_ = 78.1, p<0.001, n = 51; dark: F_(1,50)_ = 42.6, p<0.001, n = 51; Fig 3D), while the EPL response exhibited a 2-fold longer full duration at half-maximum (FDHM) than the GL response (F_(1,50)_ = 51.6, p<0.001, n = 51; Fig 3E). We also observed differences in the signal kinetics between the two layers, with the EPL response exhibiting longer rise time-to-peak (F_(1,50)_ = 16.4, p<0.001, n = 51; Fig 3F) and peak-to-trough time (F_(1,50)_ = 34.4, p<0.001, n = 51; Fig 3G). Taken together, stimulus-dependent changes in fluorescence signal within the MOB was robust and consistent in kinetics with a flavoprotein response.

**Fig 4:**
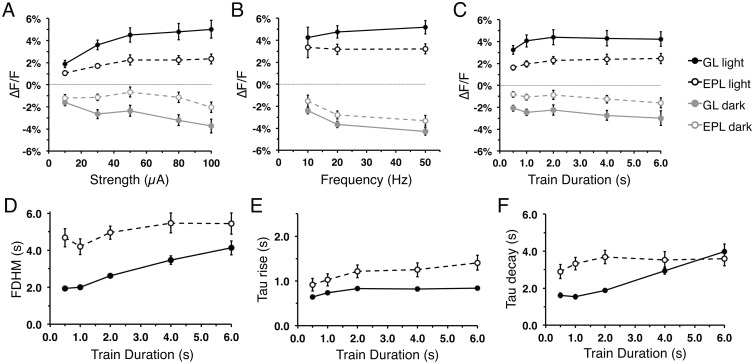
Flavoprotein signal responses vary with changes in stimulation parameters. **(A-C)** Mean flavoprotein signal amplitude of the light phase (black) and dark phase (gray) from the GL (solid) and EPL (dash) as a function of train stimulus (A) strength, (B) frequency, and (C) duration. **(D-F)** Flavoprotein GL and EPL light phase signal kinetics: (D) FDHM, (E) time-to-peak, and (F) time-to-trough as a function of train stimulus duration.

Flavoprotein signal characteristics in other brain regions are sensitive to changes in stimulation parameters [1, 7]. To further characterize and verify the flavoprotein signal in MOB slices, we performed recordings under varied stimulation strengths, train frequencies, and train durations (Fig 4). Consistent with signals seen after baseline stimulation (Fig 3), the signal amplitude remained larger in the GL than the EPL across a range of stimulation parameters. Increasing the stimulation strength from 10 μA to 100 μA while holding the other stimulation parameters constant (50 Hz, 2 s-train, 100 μs-pulse) resulted in increased amplitudes of the GL light phase (R^2^ = 0.177, F_(1,63)_ = 13.54, p<0.01, n = 13) and dark phase (R^2^ = 0.145, F_(1,63)_ = 10.65, p<0.01, n = 13), as well as in the EPL light phase amplitude (R^2^ = 0.097, F_(1,63)_ = 6.78, p<0.05, n = 6; Fig 4A). For both the GL and the EPL, the light phase amplitudes plateau at stimulations greater than 50μA. Increasing the stimulation frequency from 10 Hz to 50 Hz, while holding the other parameters constant (100 μA, 2 s-train, 100 μs-pulse) resulted in a similar increase in light and dark phase amplitudes within the GL (light: R^2^ = 0.087, F_(1,82)_ = 7.77, p<0.01, n = 6; dark: R^2^ = 0.272, F_(1,82)_ = 30.58, p<0.01, n = 21), while only the dark phase in the EPL (R^2^ = 0.143, F_(1,82)_ = 13.66, p<0.01, n = 21; Fig 4B). Increasing the stimulation train duration from 0.5 s to 6.0 s, while holding the other parameters constant (100 μA, 50 Hz, 100 μs-pulse), did not significantly alter the light and dark phase amplitudes in the GL and EPL (Fig 4C). However, increases in stimulation train duration impacted the response kinetics resulting in prolonged GL signal FDHM (R^2^ = 0.494, F_(1,83)_ = 80.96, p<0.01, n = 17; Fig 4D), GL and EPL time-to-peak (GL: R^2^ = 0.14, F_(1,83)_ = 13.47, p<0.01, n = 17; EPL: R^2^ = 0.077, F_(1,83)_ = 6.92, p = 0.01, n = 17; Fig 4E), and GL time-to-trough (R^2^ = 0.523, F_(1,83)_ = 91.0, p<0.01, n = 17; Fig 4F). Together, these results show that flavoprotein response and neuronal activity are coupled and highlight differences in response characteristics between the GL and the EPL.

### Metabolic origins of intrinsic flavoprotein fluorescent signal

**Fig 5:**
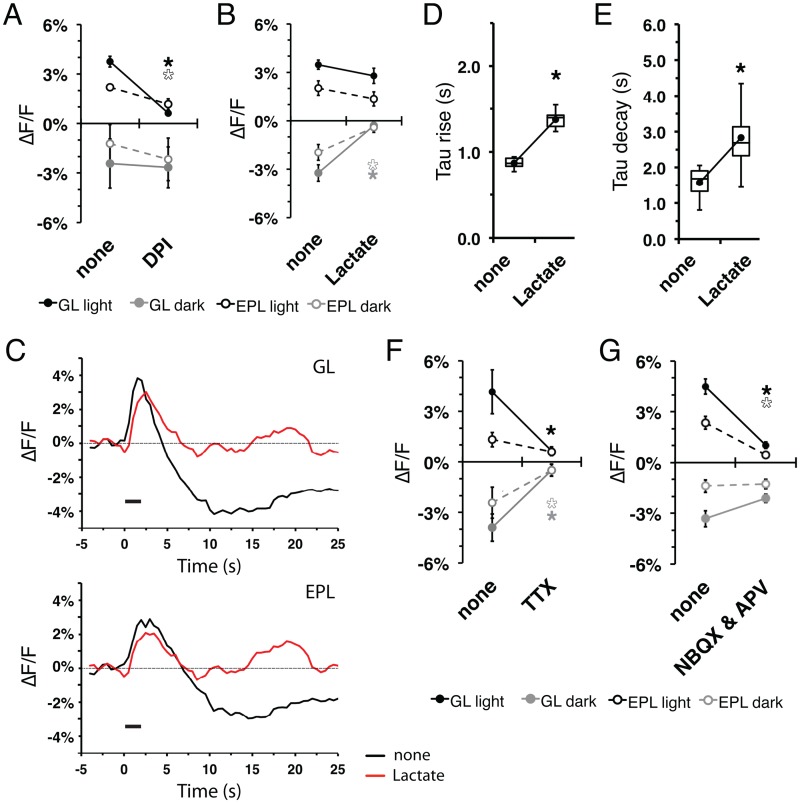
Metabolic and neuronal origins of the intrinsic flavoprotein signal response. **(A)** Mean flavoprotein signal amplitude of the light phase (black) and dark phase (gray) from the GL (solid) and EPL (dash) upon treatment with 10 μM DPI. **(B)** Mean flavoprotein signal amplitude of the light and dark phases with 15 mM lactate ACSF substitution. **(C)** Representative mean ROI traces from the activated glomerulus (top) and EPL (bottom). Line: stimulus train delivery and duration. **(D and E)** GL mean light phase time-to-peak (D) and time-to-trough (E) before and during lactate substitution. **(F)** Treatment with 10 μM TTX resulted in decreased light and dark phase amplitudes in the GL and EPL. **(G)** Mean signal peak amplitudes of light phase and dark phase in the GL and EPL upon treatment with ionotropic glutamate receptor blockers 10 μM NBQX and 50 μM AP-V.

The flavoprotein fluorescence signal results from an increase in metabolic activity within neurons [1, 5]. In other neural systems, the light and dark phases of the flavoprotein response have been attributed to mitochondrial and glycolytic activities, respectively [2]. To verify the mitochondrial origins of the flavoprotein signal light phase, we treated MOB slices with diphenyleneiodium (DPI), a mitochondrial toxin that irreversibly binds to the electron transport chain, thus preventing its activity and associated flavoprotein oxidation [20–22]. As expected, treatment with 10 μM DPI abolished the stimulus-dependent light phase (GL: F_(2,6)_ = 101.5, p<0.001, n = 3; EPL: F_(2,6)_ = 22.73, p<0.05, n = 3), while the dark phase remained unchanged in stimulated areas (Fig 5A). In order to verify the glycolytic origins of the flavoprotein signal dark phase, we treated representative slices with ACSF in which glucose was replaced with 15 mM lactate to bypass glycolytic processes while maintaining the necessary substrate for mitochondrial activity [2] (Fig 1). Lactate substitution reduced the stimulus-dependent dark phase response (GL: F_(2,6)_ = 19.23, p<0.05, n = 3; EPL: F_(2,6)_ = 8.44, p<0.05, n = 3), while the light phase response remained unchanged (Fig 5B and 5C). The kinetics of the GL light phase responses were also altered (Fig 5C–5E): lactate substitution increased the GL rise time-to-peak (F_(1,6)_ = 12.20, p<0.0001, n = 7; Fig 5D) and peak-to-trough time (F_(1,6)_ = 12.51, p<0.05, n = 7; Fig 5E). This change in kinetics suggests a partial overlap and a summation of the two signals, thus impacting the net fluorescence signal output. Therefore, the components of the biphasic flavoprotein signal response in MOB slices can be attributed to key metabolic processes, supporting the interpretation that the observed fluorescence response is due to changes in flavoprotein oxidation.

### Neuronal and post-synaptic origins of flavoprotein signal

The coupling of metabolic response and neuronal activity can be visualized due to the high metabolic demand of action potential generation [4, 23]. To a great extent, this metabolic demand results from the increased Na^+^/K^+^-ATPase and Ca^2+^-ATPase activity that follows action potential generation (Fig 1). To examine the coupling between action potential generation and flavoprotein response in this preparation, we treated MOB slices with tetrodotoxin (TTX), which irreversibly binds to and inactivates voltage-gated Na^+^ channels. Application of 2 μM TTX abolished the stimulus-dependent flavoprotein response from the GL (light: F_(1,4)_ = 12.0, p = 0.026, n = 4; dark: F_(1,4)_ = 57.7, p<0.01, n = 4), and EPL dark phase (F_(1,4)_ = 57.7, p<0.05, n = 4; Fig 5F) and a trending decrease of the EPL light phase. As with other brain regions, these observations show action potential generation is necessary for the production of stimulus-dependent flavoprotein responses.

Olfactory sensory neurons use glutamate as a neurotransmitter, and glutamate receptor antagonists block synaptic transmission from the nerve layer to the glomerular circuit [24–27]. We predicted that a cocktail of the AMPA-type glutamate receptor antagonist NBQX and the NMDA-type glutamate receptor antagonist AP-V would eliminate stimulus-dependent flavoprotein signals. Indeed, treatment with a mixture of 10 μM NBQX and 50 μM APV abolished the stimulus-dependent flavoprotein light phase response in both layers (GL: F_(2,7)_ = 70.5, p<0.001, n = 8; EPL: F_(2,7)_ = 23.8, p<0.001, n = 8); the effect on the dark phase was not statistically significant (Fig 5G). Stimulus-dependent responses were partially recovered after washout of the antagonist. Together, these results indicate that most, if not all, of the flavoprotein response observed after glomerular stimulation can be attributed to excitatory transsynaptic activation.

### Flavoprotein signal response is shaped by inhibitory input

**Fig 6:**
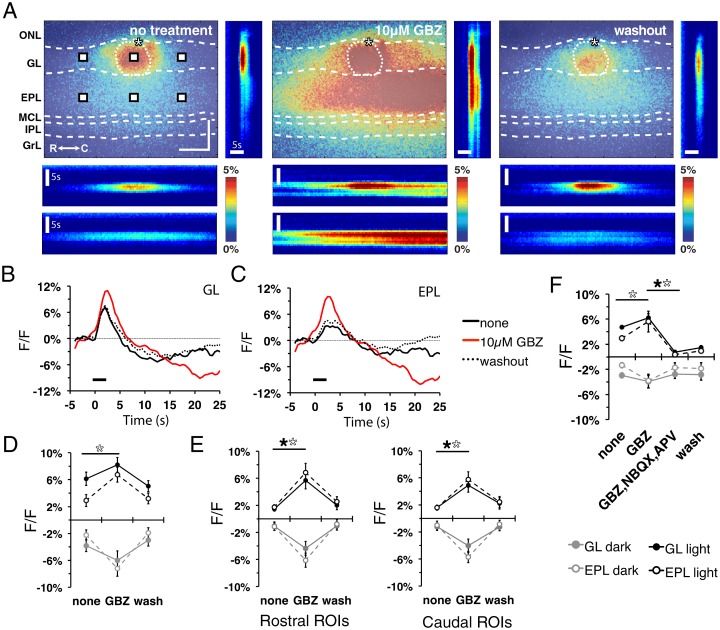
GABA_A_R antagonist enhanced flavoprotein signal response. **(A)** Stimulus-dependent flavoprotein response (left) before treatment, (middle) during bath-applied 10 μM GBZ, and (right) after wash at 1.5 s post-stimulation initiation. ONL, olfactory nerve layer; GL, glomerular layer; EPL, external plexiform layer; MCL, mitral cell layer; IPL, internal plexiform layer; GrL, granule cell layer; R, rostral; C, caudal. Asterisk, tip of the stimulating electrode. Targeted glomerulus indicated by dashed circle. GBZ treatment increased translaminar and lateral signal spread across the GL and EPL. Scale bars = 150 μm. **(B and C)** ROI traces from the (B) GL and (C) EPL from all treatment conditions indicated in (A). Traces were generated from central ROIs within the GL and EPL, as depicted in (A). Line: stimulus train delivery and duration. **(D)** Mean light phase (black) and dark phase (gray) signals from central GL (solid) and EPL (dash) ROIs exhibit increases in amplitude following GBZ treatment. **(E)** Mean light phase (black) and dark phase (gray) signal amplitudes from the rostral and the caudal ROIs of the GL (solid) and EPL (dash) as depicted in (A). Both rostral (left) and caudal (right) areas exhibited amplitude increases following GBZ treatment. **(F)** Additional treatment with 10 μM NBQX and 50 μM AP-V abolished the flavoprotein response in the GL and EPL, indicating postsynaptic activation.

Odorant information processing within the bulb is heavily modified by local, inhibitory GABAergic interneurons [27–30]. These local inhibitory interneurons include periglomerular cells within the GL as well as granule cells, which extend processes into the EPL. These interneurons produce tonic and stimulus evoked inhibition onto glomerular circuits [27, 30]. We examined how local inhibitory input shapes the flavoprotein signal and spatiotemporal spread in MOB slices. MOB slices were treated with gabazine (GBZ), a GABA_A_ receptor antagonist known to enhance postsynaptic responses [10, 14, 27] and flavoprotein signals imaged. Upon treatment with 10 μM GBZ, the amplitude and spatiotemporal spread of the stimulus-dependent flavoprotein signal were enhanced in both the GL and EPL (Fig 6A). ROI traces from the GL and EPL demonstrated increased amplitudes compared to controls (Fig 6B and 6C), showing a 2-fold increase in the EPL light phase amplitude (F_(2,5)_ = 6.7, p = 0.02, n = 6; Fig 6D). At the center of the stimulated glomerulus, signal amplitude only marginally increased, most likely due to maximal or saturated responses of the stimulated glomerulus under either condition. Examination of rostral (GL: F_(2,12)_ = 5.04, p<0.05; EPL: F_(2,12)_ = 6.57, p<0.05, n = 6) and caudal (GL: F_(2,12)_ = 7.42, p<0.05; EPL: F_(2,12)_ = 8.96, p<0.01, n = 6) neighboring areas did yield a robust enhancement of GBZ-dependent responses, suggesting a role of inhibitory input in lateral inhibition between glomeruli [27, 31] (Fig 6E). The FDHM and signal kinetics remained unchanged. The enhanced response amplitudes seen in the presence of GBZ are most likely due to unopposed glutamatergic transmission of the stimulus within the glomerular circuit. To verify this supposition, GBZ-treated slices were subsequently treated with a cocktail containing GBZ, NBQX, and AP-V. This treatment abolished flavoprotein signal response in the GL (F_(3,4)_ = 65.8, p<0.001, n = 5) and EPL (F_(3,4)_ = 7.7, p = 0.005, n = 5) relative to GBZ treatment alone (Fig 6F). Together, these results highlight the influence of inhibitory input in shaping the stimulus-dependent flavoprotein signal response and spatiotemporal spread.

### Lateral spread of stimulus-dependent flavoprotein signals

**Fig 7:**
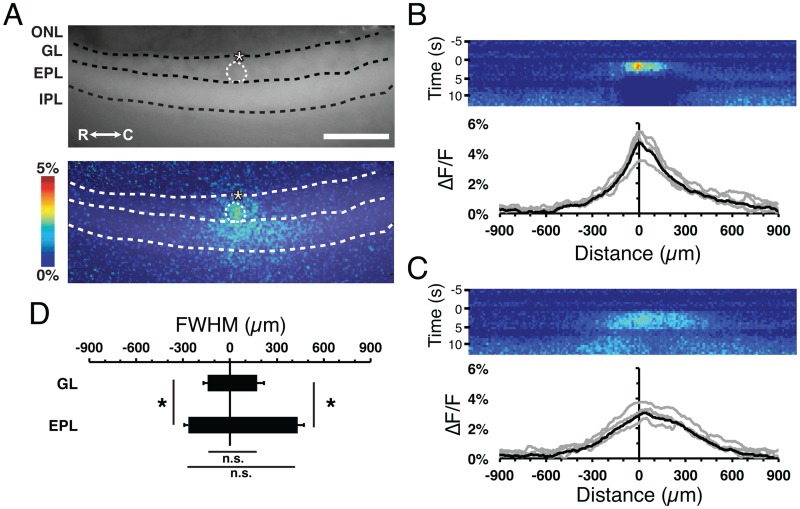
Quantification of lateral flavoprotein signal spread. **(A)** Resting light fluorescent (top) and ΔF/F flavoprotein response image at 2.5 s post-stimulus initiation (bottom) from a representative MOB slice. ONL, olfactory nerve layer; GL, glomerular layer; EPL, external plexiform layer; MCL, mitral cell layer; IPL, internal plexiform layer; GrL, granule cell layer; R, rostral; C, caudal. Asterisk, tip of the stimulating electrode. Targeted glomerulus indicated by dashed circle. Scale bar = 400 μm **(B and C)** Horizontal ROI heat maps of the (B) GL and (C) EPL taken from (A). Mean (black) and individual (gray) traces determined from the horizontal ROIs depicting lateral spread of signal averaged across the first 2.5 s of stimulus. The “0” indicates the central midline of the stimulated glomerulus, with the negative and positive values indicating rostral and caudal directions, respectively. **(D)** Mean full-width at half-maximum (FWHM) of the signal exhibited a symmetrical lateral spread perpendicular from the y-axis, where the FWHM signal spread is larger in the EPL compared to the GL.

The olfactory bulb circuit has extensive lateral connections from the long lateral dendrites of mitral/tufted neurons in the EPL, but also from the extensive short axon cell interglomerular circuit [24, 27, 32]. The spatial distribution of short axon cell projections between glomeruli has 50% of these projections extending 200 μm, and 10% projecting out to 650 μm in mouse [28]. In order to determine whether the stimulus dependent flavoprotein response amplitude correlated with the density of interglomerular projecting neurons, we assessed the complete lateral spread of flavoprotein signals at low magnification (total simultaneous field of view 2 mm, ~75% of the total length of the mouse olfactory bulb). As seen above, there was prominent translaminar and lateral spread across all the MOB layers (Figs 2A and 7A). Using horizontal ROIs within the GL and EPL, the amplitude to distance signal spread profiles were generated by averaging the maximum response across the stimulation period (Fig 7B and 7C). The spatiotemporal spread of flavoprotein signals show a symmetrical rostro-caudal distribution from the stimulated glomerulus, with no significant difference between the calculated rostral and caudal full width half maximum (FWHM) (Fig 7D). This observation is consistent with the symmetrical distribution of short axon cells within the GL [27, 28]. The calculated FWHM lateral spread in the EPL was broader than in the GL (ANOVA on Ranks, p<0.05, n = 21; Fig 7D), a finding consistent with the observation that the lateral dendrites of mitral/tufted neurons extend up to 1mm across the EPL [33]. Together, these results show that the lateral spatiotemporal spread of flavoprotein signal is consistent with the anatomic distribution of the olfactory circuit.

### Characterization of NAD(P)H signals in the main olfactory bulb

**Fig 8:**
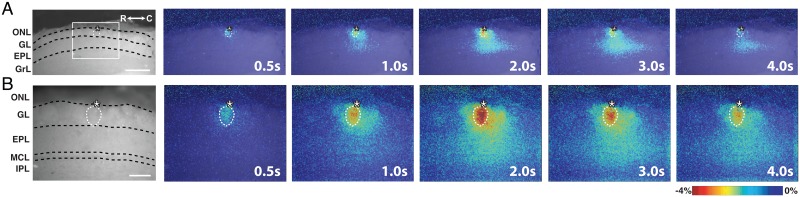
Intrinsic NAD(P)H signal response in olfactory bulb upon electrical stimulation of canonical glomerulus. **(A)** (left) Resting light fluorescence image of a horizontal MOB slice under 4x magnification. ONL, olfactory nerve layer; GL, glomerular layer; EPL, external plexiform layer; MCL, mitral cell layer; IPL, internal plexiform layer; GrL, granule cell layer; R, rostral; C, caudal. Asterisk, tip of the stimulating electrode. Targeted glomerulus indicated by dashed circle. Scale bar = 150 μm (right) Representative overlaid pseudo-colored ΔF/F images indicating the signal spread following initiation of train stimulation at 0s. **(B)** (left) Resting light fluorescent image of the demarcated region of the same slice in A at 10x magnification. Asterisk, tip of the stimulating electrode. Targeted glomerulus indicated by dashed circle. (right) Representative overlaid, pseudo-colored ΔF/F frames following initiation of train stimulation at 0s. Scale bar = 400 μm.

**Fig 9:**
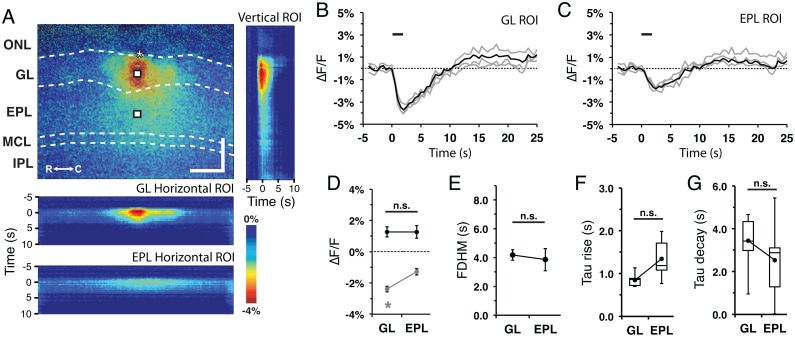
NAD(P)H signal profile in the glomerular and external plexiform layers. **(A)** (top) 10x magnification NAD(P)H image at 2.5 s post-stimulus initiation. ONL, olfactory nerve layer; GL, glomerular layer; EPL, external plexiform layer; MCL, mitral cell layer; IPL, internal plexiform layer; GrL, granule cell layer; R, rostral; C, caudal. Asterisk, tip of the stimulating electrode. Targeted glomerulus indicated by dashed circle. (right) Vertical ROI heat map across the MOB layers exhibit stimulus-dependent, trans-laminar spread over time. (bottom) Horizontal ROI heat maps from the GL and EPL exhibit stimulus dependent lateral spread over time. Scale bar = 150 μm. **(B and C)** Representative ROI individual (gray) and mean (black) traces from the activated glomerulus (B) and EPL area (C), indicated in (A). Line: stimulus train delivery and duration. **(D)** Mean signal amplitude of the initial dip (F_(11,1)_ = 57.0, p<0.001) and overshoot between the GL and EPL areas. **(E)** Mean FDHM duration between the GL and EPL. **(F and G)** NAD(P)H signal time-to-peak (F) and time-to-trough (G) between the GL and EPL.

**Fig 10:**
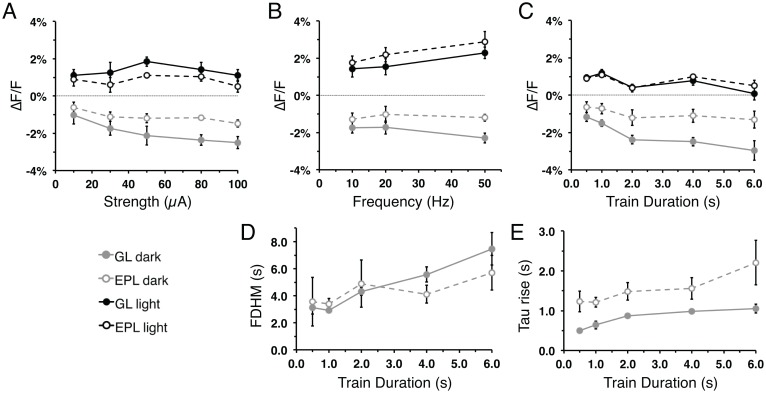
NAD(P)H signal response to changes in stimulation parameters. **(A-C)** Mean NAD(P)H signal peak (black) and trough (gray) in the GL (solid) and EPL (dash) as a function of train stimulus (A) strength, (B) frequency, and (C) duration. (A) Increasing the stimulation strength from 10μA to 100μA while holding the other stimulation parameters constant (50 Hz, 2 s train, 100 μs pulse) resulted in increased dark phase amplitudes in the GL (R^2^ = 0.324, F_(1,18)_ = 8.64, p<0.01, n = 6) and EPL (R^2^ = 0.266, F_(1,18)_ = 6.51, p<0.05, n = 6) amplitudes. (B) Increasing the stimulation frequency from 10Hz to 50Hz resulted in increased dark phase amplitudes in the EPL (R^2^ = 0.352, F_(1,14)_ = 7.61, p<0.02, n = 6). (C) Increasing the stimulation duration from 0.5s to 6.0s resulted in increased GL dark phase (R^2^ = 0.520, F_(1,18)_ = 19.49, p<0.01, n = 6) and light phase (R^2^ = 0.303, F_(1,18)_ = 7.81, p<0.02, n = 6) amplitudes. **(D)** NAD(P)H signal FDHM duration in the GL increases as a function of train stimulus duration (R^2^ = 0.267, F_(1,18)_ = 6.56, p = 0.02, n = 6). **(E)** GL (R^2^ = 0.619, F_(1,18)_ = 29.19, p<0.01, n = 6) and EPL (R^2^ = 0.287, F_(1,18)_ = 7.23, p<0.02, n = 6) signals exhibit increases in time-to-trough as a function of train stimulation duration.

NAD(P)H imaging has also been used to assess neuronal activity and map neuronal circuits [34–36]. Although NAD(P)H fluorescence is not as strong as that derived from flavoproteins, it has an advantage in that the excitation and emission wavelengths do not overlap with those of green fluorescent protein (GFP), a common genetically-encoded reporter protein. The NAD(P)H signal is inverted relative to the flavoprotein fluorescence signal, with an initial stimulus-dependent decrease in fluorescence signal followed by a prolonged increase [35]. Here we characterized stimulus-dependent NAD(P)H signals in MOB slices. Electrical stimulation of the glomerular circuit produced robust NAD(P)H signals in MOB slices (Fig 8). Similar to intrinsic flavoprotein imaging, the stimulus-dependent NAD(P)H response displayed translaminar and lateral spatiotemporal signal spread (Fig 9A). Consistent with other brain regions [37–39], ROI traces from the GL and EPL displayed stimulus-dependent decrease in fluorescence signal followed by a prolonged signal elevation (Fig 9B and 9C). Signal amplitudes of the initial dark phase were larger in the GL than in the EPL (F_(1,11)_ = 57.0, p<0.001, n = 12; Fig 9D), but exhibited no differences were seen in the signal kinetics (FDHM, rise time, decay tau; Fig 9E–9G). The NAD(P)H response was also sensitive to changes in stimulation strength, frequency, and duration (Fig 10A–10C). The kinetics of the NAD(P)H signal in the GL was affected by changes in the stimulus train duration, resulting in increased GL FDHM (R^2^ = 0.267, F_(1,18)_ = 6.56, p = 0.02, n = 6), and time-to-peak in the GL (R^2^ = 0.619, F_(1,18)_ = 29.19, p<0.01, n = 6) and EPL (R^2^ = 0.287, F_(1,18)_ = 7.23, p<0.02, n = 6; Fig 10D and 10E). Similar to our results with flavoprotein imaging, the lateral spatiotemporal spread of the NAD(P)H signal was broader in the EPL (F_(3,13)_ = 51.7, p<0.001, n = 14) and insensitive to changes in stimulus parameters (data not shown). All together, the stimulus-dependent response characteristics of the NAD(P)H and flavoprotein signals in MOB slices were remarkably similar.

## Discussion

Here we provide a detailed characterization of flavoprotein and NAD(P)H fluorescence signals in horizontal MOB slices following electrical train stimulation of single glomeruli. Both intrinsic signals generated the biphasic response profiles and sensitivity to stimulus parameter changes observed in other brain regions [1, 2, 35, 37, 40]. Intrinsic flavoprotein signals exhibited loss of response in the presence of mitochondrial toxin, metabolic substrate substitution, and ionic glutamate receptor antagonists, confirming the metabolic and transsynaptic nature of these signals. Both signals exhibited a spatiotemporal spread within the olfactory bulb consistent with the microcircuit organization of the MOB. Signal amplitude and spatiotemporal spread were enhanced in the presence of GABA_A_ receptor antagonists, indicating baseline responses seen in untreated slices are heavily shaped by inhibitory inputs and emphasizing the importance of inhibitory inputs in shaping the transmission of olfactory information in the MOB.

### Technical considerations

This study and others demonstrate the applicability of intrinsic fluorescence imaging to examine the functional neuronal circuitry. As with any functional imaging method the exact placement of the stimulating electrode relative to the organization of the pathway impacts the amplitude and spatiotemporal profile of the signal [14, 41]. In the olfactory system, superficial positioning of the stimulus electrode in the ONL of the MOB will generate post-synaptic circuit activation. However, in this position, signal spread is broad and primarily extended toward the caudal end of the MOB (data not shown) consistent with stimulation of fibers of passage en-route to caudal glomeruli leading to the activation of multiple glomeruli. We and others have found placement of a micro-theta stimulating electrode must be on the bundles of axons going to the target glomerulus to result in stimulation of a single, or several, glomeruli and associated post-synaptic circuitry.

Robust and repeatable intrinsic fluorescence signal responses in other brain regions depend on a specific range of stimulus parameters [1, 7]. As seen in other brain regions, the generation of flavoprotein and NAD(P)H signals in the olfactory bulb requires the delivery of a train stimulus [7, 35], which is hypothesized to sufficiently increase the metabolic demand in postsynaptic cells to enable robust detection [1, 5]. However, some brain regions have produced intrinsic signals following a single pulse stimulation, albeit of much smaller amplitudes [1]. We found that a single pulse stimulation is insufficient to generate consistent and robust intrinsic flavoprotein and NAD(P)H fluorescence signal response. The olfactory circuitry exhibits a high level of spontaneous activity driven by the intrinsic bursting of external tufted cells, and it may be that responses to a single stimulation do not produce sufficient additional metabolic demands to be detectable with flavoprotein and NAD(P)H fluorescence imaging. However, within the limitation of requiring more than a single stimulus event, intrinsic metabolic imaging is robustly detectable.

### Representation of the glomerular circuit

The results in this study outline the applicability of metabolic imaging for examining the functional circuitry of the MOB without the use of complex equipment, external dyes, or genetically modified mice. When compared to other intrinsic imaging techniques, such as MRI, PET, or hemodynamic responses, intrinsic fluorescence imaging has the flexibility to be performed *in vivo* or *in vitro* using a standard epifluorescence microscope. This accessibility has allowed previous studies to generate cortical sensory maps with more accuracy than hemodynamic imaging [6, 42]. Intrinsic fluorescence imaging has been used in the olfactory system to examine *in vivo* odor-evoked responses on the surface of the MOB [43]. However, neither intrinsic flavoprotein or NAD(P)H imaging is efficient *in vivo*, in part because of the high absorbance of the GL [44]. This limitation is mitigated in horizontal MOB slices, where much of the MOB circuitry is preserved and translaminar optical responses can be observed.

The lateral spatiotemporal spread of metabolic imaging signals within the MOB is consistent with the anatomic distribution of neurons/connectivity within the MOB circuitry. Based on tracing studies, mitral/tufted cells associated with a single glomerulus display a broader anatomical distribution and long-range lateral processes within the EPL [32, 45]. The distance of periglomearular/tufted cell bodies from their glomerulus of origin is short, an average distance of 69 μm, with some cells extending as far as 150 μm [32]. However, the long range short axon cell distribution shows 50% of short axon cells found at ~400 μm from label-injected glomeruli [27, 28]. Our observed GL signal spread falls comfortably in the range of anatomic projections within the glomerular layer. EPL FWHM signal spread (300–400 μm) exceeded earlier observations of mean cell body distances for projections neurons associated with a glomerulus, but within the distribution of their lateral dendrites. Previous studies reported that the average distance of mitral/tufted cell bodies from the glomerular midline is 111–116 μm [32], with 80% of the cells found within 111 μm [33, 46]. Mitral/tufted cell lateral dendrites, however, project approximately 750 μm from the cell bodies [45], with some extending as far as 1500 μm across the MOB [46–48]. This suggests lateral dendrite engagement from glomerular stimulation produces enough neural activity to elicit a significant metabolic demand. Given the significant spread of stimulus-dependent flavoprotein/NAD(P)H fluorescence in both the GL and EPL, the majority of the intrinsic signal likely originated from the neuronal processes of postsynaptic juxtaglomerular and mitral/tufted cells. This is consistent with the observation that neuronal processes have high mitochondrial densities and are highly metabolically active [4, 49].

We observed differences in intrinsic fluorescence signal profiles between the GL and EPL. The higher amplitude signals from the GL could be due to the combination of higher cellular density and potentially higher activity yielding higher energy demand. Individual glomeruli are densely packed with processes from the terminal nerve fibers, juxtaglomerular cells, mitral and tufted cell apical dendrites and cell bodies of juxtaglomerular neurons [50]. Estimations of glomerular neuropil indicate that approximately 30% of a given glomerulus neuropil is occupied by presynaptic components [13], while another 45% was attributed to postsynaptic processes of juxtaglomerular, mitral, and tufted cells [51]. The interglomerular space is almost entirely somatic with sparse neuropil [16, 52]. The higher energy demand suggested by our imaging was consistent with the increased blood flow seen in the GL during odorant exposure [53, 54]. We provide direct evidence of such a higher metabolic load consistent with the inference from the higher vascular density.

In addition to the gross differences in the composition and energy consumption of MOB layers, variations in the intrinsic signal profiles may be attributed to differences in the subcellular responses and cell type. Optical recordings of Ca^2+^ signals from different compartments of mitral cells exhibit variations in response amplitudes [55–58]. The apical dendritic tuft and shaft exhibited higher response amplitude than the cell soma, which suggests a higher ion flux and consequential ion pump energy demand. Along with subcellular variation in intrinsic signal response, non-neuronal cell types could potentially contribute to the overall intrinsic response due to the presence of flavoproteins and NAD(P)H in all cells. Detailed characterizations of stimulus-dependent flavoprotein responses in cerebellar slices have attributed the initial light phase and the later dark phase primarily to neurons and glia, respectively [1, 2, 59]. The later dark phase was abolished when cerebellar slices were treated with fluoroacetate, an inhibitor of glial metabolism [59]. Characterizations of intrinsic NAD(P)H signals within hippocampal slices have reached similar conclusions [39]. As such, we emphasize the use of initial flavoprotein light phase assessing the spatiotemporal spread in neuronal activity.

## Conclusions

There are several benefits to the use intrinsic flavoprotein and NADH imaging to understand the circuitry of the MOB. The method is largely accessible and can be applied to either *in vitro* or *in vivo* preparations with intact circuitry. The observed stimulus-dependent intrinsic signals are predominantly due to postsynaptic responses and consistent with our understanding of the glomerular circuit anatomy and cellular distribution. Intrinsic fluorescence imaging also allows for the direct observation of neurometabolism that has been also correlated with synaptic plasticity [60, 61]. With advancements in microscopy and multiphoton imaging, it may be possible to determine how subcellular metabolic responses relate to the electrophysiological changes seen in activated neurons. Metabolic demand, oxidative stress, and overall cellular energy homeostasis are emerging as critical issues in neuronal survival in models of traumatic brain imaging.

Overall, intrinsic fluorescence imaging can be used to rapidly address the organization and connectivity of the MOB circuitry and to assess the flow of olfactory information through the MOB [62]. The accessibility of the imaging technique makes it an ideal tool to complement other physiological methodologies and open new avenues of research.
